# The Polar Fox Lagoon in Siberia harbours a community of Bathyarchaeota possessing the potential for peptide fermentation and acetogenesis

**DOI:** 10.1007/s10482-022-01767-z

**Published:** 2022-08-10

**Authors:** Tom Berben, Franco Forlano Bó, Michiel H. in ‘t Zandt, Sizhong Yang, Susanne Liebner, Cornelia U. Welte

**Affiliations:** 1 Department of Microbiology, Radboud Institute for Biological and Environmental Sciences, Radboud University, Nijmegen, The Netherlands; 2 Netherlands Earth System Science Centre, Utrecht University, Utrecht, The Netherlands; 3 Section Geomicrobiology, GFZ German Research Centre for Geosciences, Potsdam, Germany; 4 Cryosphere Research Station On the Qinghai-Tibet Plateau, State Key Laboratory of Cryospheric Sciences, Northwest Institute of Eco-Environment and Resources, Chinese Academy of Sciences, Lanzhou, China; 5 Institute of Biochemistry and Biology, University of Potsdam, Potsdam, Germany

**Keywords:** Bathyarchaea, Siberia, Thermokarst, Peptide fermentation

## Abstract

Archaea belonging to the phylum Bathyarchaeota are the predominant archaeal species in cold, anoxic marine sediments and additionally occur in a variety of habitats, both natural and man-made. Metagenomic and single-cell sequencing studies suggest that Bathyarchaeota may have a significant impact on the emissions of greenhouse gases into the atmosphere, either through direct production of methane or through the degradation of complex organic matter that can subsequently be converted into methane. This is especially relevant in permafrost regions where climate change leads to thawing of permafrost, making high amounts of stored carbon bioavailable. Here we present the analysis of nineteen draft genomes recovered from a sediment core metagenome of the Polar Fox Lagoon, a thermokarst lake located on the Bykovsky Peninsula in Siberia, Russia, which is connected to the brackish Tiksi Bay. We show that the Bathyarchaeota in this lake are predominantly peptide degraders, producing reduced ferredoxin from the fermentation of peptides, while degradation pathways for plant-derived polymers were found to be incomplete. Several genomes encoded the potential for acetogenesis through the Wood-Ljungdahl pathway, but methanogenesis was determined to be unlikely due to the lack of genes encoding the key enzyme in methanogenesis, methyl-CoM reductase. Many genomes lacked a clear pathway for recycling reduced ferredoxin. Hydrogen metabolism was also hardly found: one type 4e [NiFe] hydrogenase was annotated in a single MAG and no [FeFe] hydrogenases were detected. Little evidence was found for syntrophy through formate or direct interspecies electron transfer, leaving a significant gap in our understanding of the metabolism of these organisms.

**Supplementary Information:**

The online version contains supplementary material available at 10.1007/s10482-022-01767-z.

## Introduction

The Bathyarchaeota are an archaeal phylum whose members are globally distributed in anoxic marine and freshwater sediments, and soils (Zhou et al. 2018). Their link with biogeochemical carbon cycles, together with the predominance of Bathyarchaeota in cold subsurface environments, points to an important role for Bathyarchaeota in the emission of greenhouse gases such as carbon dioxide (CO_2_) and methane (CH_4_) in a warming world. This is particularly concerning in the Arctic permafrost, which stores approximately 1300 Pg of carbon (Hugelius et al. 2014) which is rapidly becoming bioavailable through thermokarst formation (in ’t Zandt et al. 2020; Turetsky et al. 2020). Briefly, thawing permafrost destabilizes sediments, leading to the formation of thermokarst lakes. Taliks, regions of thawed sediment within permafrost, can form below these lakes and drive their expansion. Thermokarst landscapes are now widespread in the Arctic regions of Alaska, Canada and Siberia (Angelopoulos et al. 2020; Olefeldt et al. 2016). Thermokarst lakes located near seas or bays may become part of these due to sea level rise and/or coastal erosion, leading to mixing of fresh and saline waters. In intermediate stages, a lagoon may be formed which allows for seasonal flow of seawater into the thermokarst lake (Jenrich et al. 2021). The inflow of more saline water influences the freeze/thaw dynamics in these lagoons in complex ways. Angelopoulos et al. (2020) showed that the Polar Fox Lagoon in Siberia is at an equilibrium point where accumulation of colder, more saline water at the bottom of the lake allows the underlying talik to refreeze in winter. Even a slight raise in temperature, however, is predicted to allow saline water to penetrate the soil, thus lowering its freezing point and keeping the talik thawed permanently. The lake water itself is subject to winter freezing and the formation of ice eventually closes the channel to Tiksi Bay, isolating it from the sea. Methane is trapped in the ice as it is formed, as well as below the ice, where it is available for biological oxidation or release to the atmosphere upon ice thawing (Heslop et al. 2020; Spangenberg et al. 2021).

Initially described as the “Miscellaneous Crenarchaeotal Group” (MCG) in a survey of 16S rRNA gene sequences from a hot spring located in Yellowstone National Park, USA (Barns et al. 1996), Bathyarchaeota have been shown to be the predominant archaeal representative in cold and anoxic marine sediments (Lloyd et al. 2013). The rapid increase of published metagenomic datasets over the past decade has revealed that Bathyarchaeota occur in a wide range of habitats besides seafloor sediments, e.g. in hydrothermal vents (He et al. 2016), mangroves (Pan et al. 2019), urban canal systems (Pelsma et al. 2022) and thermokarst lakes (de Jong et al. 2018; Winkel et al. 2018). These archaea are implicated in the global carbon cycle and may have a large impact on carbon dioxide and methane emissions into the atmosphere. Unfortunately, no pure cultures of Bathyarchaeota currently exist so that we have an incomplete understanding of their metabolic potential, however, very recently, new enrichment strategies have been explored, resulting in several co-cultures of a Bathyarchaeon with a bacterial partner (Hu et al. 2021). Several previous studies have set out to create a classification of Bathyarchaeal lineages (Fillol et al. 2016; Kubo et al. 2012), which were unified into a set of 25 distinct lineages in a review by Zhou et al. (2018). The Genome Taxonomy Database (GTDb), an endeavour to create a unified and systematic taxonomy of Bacteria and Archaea based on phylogenomic analysis, currently proposes that the Bathyarchaeota be treated as the class Bathyarchaeia, rather than as a phylum. The class Bathyarchaeia is placed within the phylum Thermoproteota, alongside the classes Korarchaeia, Methanomethylicia, Thermoproteia and Nitrososphaeria (Parks et al. 2018, 2021). As of November 2021, the class Bathyarchaeia in GTDb contains 173 MAGs, which are further classified into seven orders.

Despite the lack of cultured representatives, the metabolic potential of Bathyarchaeota has been extensively explored through metagenomics, particularly metagenome-assembled genomes (MAGs), and single-cell genomics (Evans et al. 2015; He et al. 2016; Lloyd et al. 2013; Meng et al. 2014). Numerous studies have associated Bathyarchaeota with a wide array of different metabolisms: energy could be conserved through fermentative degradation of detrital proteins (Lloyd et al. 2013), fatty acids (Evans et al. 2015), and aromatics such as protocatechuate (Meng et al. 2014). The fate of the liberated reducing equivalent is more elusive and probably differs between the bathyarchaeotal lineages. Some Bathyarchaeota possess genes encoding methyl- or acyl-CoM reductase and electrons liberated from the catabolism of aforementioned substrates could potentially be used to reduce methyl- or longer chain alkyl-groups derived from various substrates to methane or higher chain alkanes (Borrel et al. 2019; Laso-Pérez et al. 2019; Wang et al. 2019). However, with the current knowledge it cannot be excluded that Bathyarchaeota use the reverse methanogenesis/ethanogenesis pathway to oxidize methane or higher chain alkanes instead (Borrel et al. 2019; Evans et al. 2015; Hahn et al. 2021; Laso-Pérez et al. 2016, 2019). Other studies have demonstrated that several Bathyarchaeotal lineages appear to possess the genes necessary for homoacetogenesis, a lifestyle that was thus far associated solely with bacteria (He et al. 2016). One study demonstrated that the addition of lignin, a refractory plant-derived polymer containing methoxy groups, stimulated the chemoorganoautotrophic growth of the Bathy-8 lineage (Yu et al. 2018).

To study the potential role of Bathyarchaeota in methane formation in the Polar Fox Lagoon, we have studied 19 MAGs recovered from a metagenomic dataset of sediment samples taken from the lagoon in April 2017. We present a reconstruction of the metabolic potential of these organisms.

## Materials and methods

### Sample collection

Polar Fox Lagoon is located on the Bykovsky Peninsula, southeast of the Lena Delta in the Buor-Khaya Gulf of the Laptev Sea in northeastern Siberia, Russia. The lagoon is a nearly-closed system with a wide, shallow and winding channel supplying the water body with water from Tiksi Bay during the summer, with large seasonal variation in salinity and ion concentrations such as sulfate and chloride (Angelopoulos et al. 2020; Spangenberg et al. 2021). A 6.1 m long composite core was recovered during a drilling campaign in April 2017 using a hammer-driven 60 mm Niederreiter piston corer (UWITEC™) from overlapping core sections retrieved in 3-m-core barrels. The core consisted of unfrozen sediment at time of sampling. After retrieval, the cores were cut into 10 cm liner segments on-site. Sediment plugs were obtained from their edges using cut, sterile syringes and placed into sterile 15 ml falcon tubes which were kept frozen until DNA extraction. For a detailed analysis of physiochemical properties of the core samples see Yang et al. (2022).

### DNA extraction and sequencing

DNA was extracted from six samples along the composite core in duplicates using the PowerSoil DNA (Qiagen; replicate 1) and the FastSpin DNA (MP Biomedicals; replicate 2) extraction kits. DNA concentrations were checked through gel electrophoresis and quantified on a 2.0 Qubit Fluorometer (ThermoFisher Scientific, Darmstadt, Germany) according to the protocol of the DNA High Sensitive and Broad Range Assay Kit (ThermoFisher, Berlin, Germany). DNA was sent for paired-end sequencing at GATC Biotech (now Eurofins Scientific, Constance, Germany) on an Illumina Hiseq 2500 system.

Samples for DNA extraction were selected based on the δ^13^C-CH_4_ profile measured in 10 cm sections of the obtained sediment cores (Yang et al. 2022). The bottom section was defined by a δ^13^C-CH_4_ profile around −80, whereas the top profile showed a δ^13^C-CH_4_ profile ranging between −55 and −40. The transition layer was defined as the zone in which a decrease in the δ^13^C-CH_4_ values was observed (Table 1).

**Table 1:** Overview of the top section, transition layer and bottom section of the Polar Fox Lagoon sediment, based on the δ^13^C-CH_4_ profile. Within the top section and bottom section the δ13C-CH4 profiles were highly similar and samples were pooled for further analysis. For the transition layer, a higher sample resolution was chosen

Section of core	Core section(s) in cm from top of core sample, respective core	Real depth below sediment surface (cm)	Corresponding SRA sample names
Top section	30–40 cm (core 1) 90–100 cm (core 1) 120–130 cm (core 1)	30–40 90–100 120–130	1_F_500mg_1 1_PS_500mg_1
Transition layer #1	180–190 cm (core 1)	180–190	2_F_500mg_1 2_PS_500mg_1
Transition layer #2	210–220 (core 1)	210–220	3_F_500mg_1 3_PS_500mg_1
Transition layer #3	0–10 cm (core 2)	240–250	4_F_500mg_1 4_PS_500mg_1
Bottom section	30–40 cm (core 2) 150–160 cm (core 2) 30–40 cm (core 3)	270–280 390–400 440–450	5_F_500mg_1 5_PS_500mg_1
Suspected permafrost section	120–130 cm (core 3)	530–540	6_F_500mg_1 6_PS_500mg_1

Sediment samples were taken aseptically from core subsamples stored at −18 °C. For the top section and bottom section, samples were obtained by pooling equal amounts of sediment of each subsample, based on weight. The pooled samples were mixed thoroughly.

DNA was extracted in duplicate per sample using two different extraction methods. For the first method DNA was extracted using the Power Soil DNA Isolation Kit (Qiagen, Venlo, the Netherlands) following the manufacturer’s instructions with the following modifications. A total of 500 mg soil was added to each extraction tube. PowerBead Tubes were inserted in a vortex tube holder and vortexed at maximum speed for 10 min. Final DNA samples were eluted in two steps with 35 µL sterile Milli-Q, obtaining an end volume of 70 µL.

For the second method DNA was extracted using the FastDNA™ Spin Kit for Soil (MP Biomedicals SARL, Illkirch-Graffenstaden, France) following the manufacturer’s instructions with the following modifications. A total of 500 mg soil was added to each extraction tube. Vortexing of samples was done in two steps of 1 min and cooling down for 1 min on ice in between. After vortexing, samples were centrifuged for 10 min at 14,000 xg. After adding binding matrix, tubes were inverted by hand for 2 min and then settled for 3 min. Samples were eluted in 70µL DNAse-free water.

DNA quantity was measured fluorometrically by using the Qubit dsDNA HS Assay Kit (Invitrogen, Thermo Fisher, Carlsbad, CA, USA) according to the manufacturer’s instructions. Per extraction method, the replicate with the highest DNA yield was selected for metagenome sequencing. DNA samples were stored at −18 °C until further analysis.

Metagenome sequencing was performed using the metagenomics sequencing service of Eurofins Genomics Europe Shared Services GmbH (Eurofins Genomics, Konstanz, Germany). Quality controls were performed by Eurofins Genomics and included DNA quantity and integrity measurements.

### Assembly and binning

An in-house bioinformatics pipeline for metagenome assembly and binning was used, as described in in ‘t Zandt et al. (2019). Briefly, quality assessment and subsequent trimming/filtering of sequencing reads was performed using BBDuk. Co-assembly of the remaining reads was performed using metaSPAdes 3.11.1 (Nurk et al. 2017). Five different algorithms were used to bin contigs larger than 1,500 bp (Alneberg et al. 2014; Graham et al. 2017; Kang et al. 2015; Lu et al. 2017; Wu et al. 2016) and DAS tool was used to construct a consensus metagenome (Sieber et al. 2018).

### Analysis of metagenome-assembled genomes

CheckM 1.0.11 was used to estimate the completeness, contamination and strain heterogeneity of each of the nineteen MAGs (Parks et al. 2015), using the lineage_wf mode at default settings. GTDB-tk 0.3.2 was used to generate a phylogenomic classification of the genomes based on a combination of metrics derived from single-copy conserved gene phylogeny and average nucleotide identity (Chaumeil et al. 2020; Parks et al. 2021). We used the classify_wf at default settings. Prokka was used for automated annotation, using the ‘-kingdom Archaea’ flag, but default settings otherwise. DRAM (Shaffer et al. 2020) was used to gain an initial impression of the metabolic capabilities of the Bathyarchaea and to find interesting pathways to study in more detail. HydDB (Søndergaard et al. 2016) was used to classify the catalytic subunits of the hydrogenases found by DRAM.

The MEROPS database (Rawlings et al. 2018) was used to identify putative proteases/peptidases in all MAGs. For every MAG, all protein sequences predicted by Prokka were blasted against the MeropsScan library (a subset of the full database specifically designed to facilitate classification) at an e-value threshold of 10^–4^, the same as used by the original implementation of MEROPS batch blast, which is unfortunately no longer available (Rawlings and Morton 2008). Query sequences were then linked to the MEROPS family of their BLASTP hits. Sequences that gave multiple hits against the MeropsScan library were manually checked, but in all cases all hits led to the same classification. The sequences identified as peptidases by this process were then collected and used as input for SignalP 5.0 (Almagro Armenteros et al. 2019), to identify export signals.

The dbCAN2 server (Zhang et al. 2018) was used to identify CAZymes and CAZyme gene clusters. The protein fasta files and corresponding GFF files produced by Prokka were used as input. The search was run using default parameters. To check the potential for lignin degradation, specifically the degradation of methoxy groups in lignin, we manually blasted MtoABCD from *Methermicoccus shengliensis*, a group of enzymes for which this activity has been shown experimentally (Kurth et al. 2021), against the Bathyarchaeal MAGs, at an e-value threshold of 10^–4^. The accession numbers for the query sequences are: WP_042685937.1 (MtoA), WP_042685515.1 (MtoB), WP_042685521.1 (MtoC), and WP_042685513.1 (MtoD).

### Phylogenetic analysis

To classify the annotated (partial) 16S rRNA gene sequences recovered from the PFL MAGs, we retrieved the sequence collection used by Zhou et al. (2018) from https://github.com/ChaoLab/Bathy16Stree. We then added the five new sequences, aligned using SINA 1.2.11 with the SSU database version 138. Columns with more than 50% gaps were removed using trimAl (Capella-Gutierrez et al. 2009). The maximum-likelihood tree was generated by RAxML 8.2.10 using rapid bootstrapping (500 replicates) with the GTR + GAMMA model. We manually checked the tree topology to ensure it corresponded to that presented by Zhou et al. Subgroup classification for the PFL sequences was subsequently inferred from their placement within the tree.

## Results and discussion

### Statistics and phylogenetic and –genomic classification of Bathyarchaeotal metagenome-assembled genomes from the Polar Fox Lagoon

Metagenome sequencing, assembly and binning on the Polar Fox Lagoon sediment core samples produced 164 MAGs. A total of 126 MAGs (78%) were of bacterial origin, being classified as belonging to diverse phyla such as the Chloroflexi, Planctomycetes, Actinobacteria and Proteobacteria. Of the 38 archaeal MAGs, 19 belonged to the Bathyarchaeota and the other 19 mostly to the Thermoplasmatota and Halobacterota. In the present study, we were interested in the metabolic potential encoded for by the Bathyarchaeotal MAGs.

### Properties of Bathyarchaeotal metagenome-assembled genomes from the Polar Fox Lagoon

Table 2 shows an overview of the properties of the Bathyarchaeotal MAGs, including genome size, GTDB classification down to the genus level (if available) and CheckM statistics. A CheckM completeness score of > 90% was set for downstream analysis and 6 MAGs met this criterion. Of these, MAG004 also showed high contamination and strain heterogeneity scores. Five MAGs had completeness scores < 70%, making them poor candidates for metabolic reconstruction, as it is impossible to infer from the present data alone whether any missing gene is actually absent, or merely unassembled or unbinned.

**Table 2:** GTDB classification and CheckM statistics of 19 Bathyarchaeotal MAGs recovered from the Polar Fox Lagoon metagenome dataset. Italic rows indicate MAGs that contain an annotated 16S ribosomal DNA sequence. Data for MAGs belonging to other phylogenetic groups are included in Supplementary table S1﻿

Genome	# contigs	GTDb classification				CheckM statistics		
		Class	Order	Family	Genus	Completeness	Contamination	Strain heterogeneity
MAG002	240	Bathyarchaeia	B26-1	UBA233	20-14-0-80-47-9	99.07	8.88	8.33
*MAG004*	*513*	*Bathyarchaei*a	*B26-1*	*UBA233*	*20-14-0-80-47-9*	*97.2*	*22.74*	*21.21*
MAG011	257	Bathyarchaeia	B26-1	UBA233	20-14-0-80-47-9	95.33	6.07	14.29
*MAG014*	*217*	*Bathyarchaeia*	*40CM-2-53-6*			*94.63*	*4.67*	*16.67*
MAG019	117	Bathyarchaeia	40CM-2-53-6			92.72	5.83	12.5
MAG033	488	Bathyarchaeia	B26-1	UBA233		91.12	9.97	33.33
MAG048	609	Bathyarchaeia	TCS64	TCS64		87.86	12.37	57.89
*MAG060*	*500*	*Bathyarchaeia*	*RBG-16-48-13*			*86.14*	*9.66*	*6.25*
MAG062	1118	Bathyarchaeia	RBG-16-48-13			85.37	21.77	7.84
MAG066	457	Bathyarchaeia	B26-1	BA1		84.95	14.54	24
MAG095	359	Bathyarchaeia	B26-1	UBA233	20-14-0-80-47-9	76.17	5.21	62.5
MAG097	658	Bathyarchaeia	B26-1	UBA233	AD8-1	76.04	20.31	54.55
MAG098	331	Bathyarchaeia	B26-1	UBA233	20-14-0-80-47-9	75.24	8.74	33.33
MAG102	500	Bathyarchaeia	B26-1	BA1	BIN-L-1	73.82	15.53	27.27
MAG107	619	Bathyarchaeia	TCS64	TCS64	RBG-16-57-9	72.83	7.86	72.73
MAG114	691	Bathyarchaeia	40CM-2-53-6	RBG-13-38-9		68.7	18.07	5.88
MAG117	526	Bathyarchaeia	B26-1	UBA233		67.98	19.09	7.41
*MAG121*	*442*	*Bathyarchaeia*	*B26-1*	*UBA233*	*20-14-0-80-47-9*	*66.36*	*15.89*	*13.79*
MAG125	448	Bathyarchaeia	RBG-16-48-13			63.92	4.75	40
MAG137	275	Bathyarchaeia	B26-1	UBA233	PALSA-986	55.06	6.54	0

### Phylogenomic and –genetic analysis of Bathyarchaeotal MAGs from the Polar Fox Lagoon

GTDB-tk assigned the MAGs to four distinct orders, of which B26-1 was the most abundant (12 out of 19), followed by 40CM-2-53-6 and RBG-16-48-13 (3 out of 19 each), and TC64S (2 out of 19). This corresponds to the overall number of genomes in these orders in the GTDb, where B26-1 represents over half of the Bathyarchaeia (98 out of 173), followed by 40CM-2-53-6 (41 out of 173).

A more fine-grained phylogenetic classification scheme of the Bathyarchaeota based on 16S rRNA gene phylogeny and concatenated ribosomal protein analyses has been established previously, currently recognizing 25 distinct sub-groups (Zhou et al. 2018). Unfortunately, we were only able to annotate (partial) 16S rRNA gene sequences for four of the MAGs presented in this study. Following the protocol established by Zhou et al. (see materials and methods for a brief description), these genomes were classified as follows: MAG004, Bathy-5b; MAG014, two 16S rRNA gene sequences that both cluster with Bathy-18; MAG060, Bathy-17; and MAG121, Bathy-17. Bathy-5b is strongly associated with freshwater habitats, whereas Bathy-17 contains a majority of sequences retrieved from saline environments, and Bathy-18 is associated with freshwater, saline and even hypersaline environments. The transition that the Polar Fox Lagoon (PFL) is undergoing from an isolated freshwater lake, to a lagoon that is intermittently connected to Tiksi Bay and, upon further coastal erosion, eventually to being part of Tiksi Bay, means that the salinity is likely to rise over time (Angelopoulos et al. 2020; Spangenberg et al. 2021). While Tiksi Bay itself is brackish, increased salt concentrations may have a negative impact on Bathy-5b group archaea, while favouring other subtypes more strongly associated with a saline habitat. Salt concentrations may also vary within a single lake or lagoon, as stratification leads to higher concentrations near the sediment (Heslop et al. 2020). Previous research on the Pearl River Estuary in China showed that salinity is a major factor determining the composition of the Bathyarchaeotal community (Zou et al. 2020).

### All MAGs contain protein degradation pathways without extracellular peptidases

**Fig. 1:**
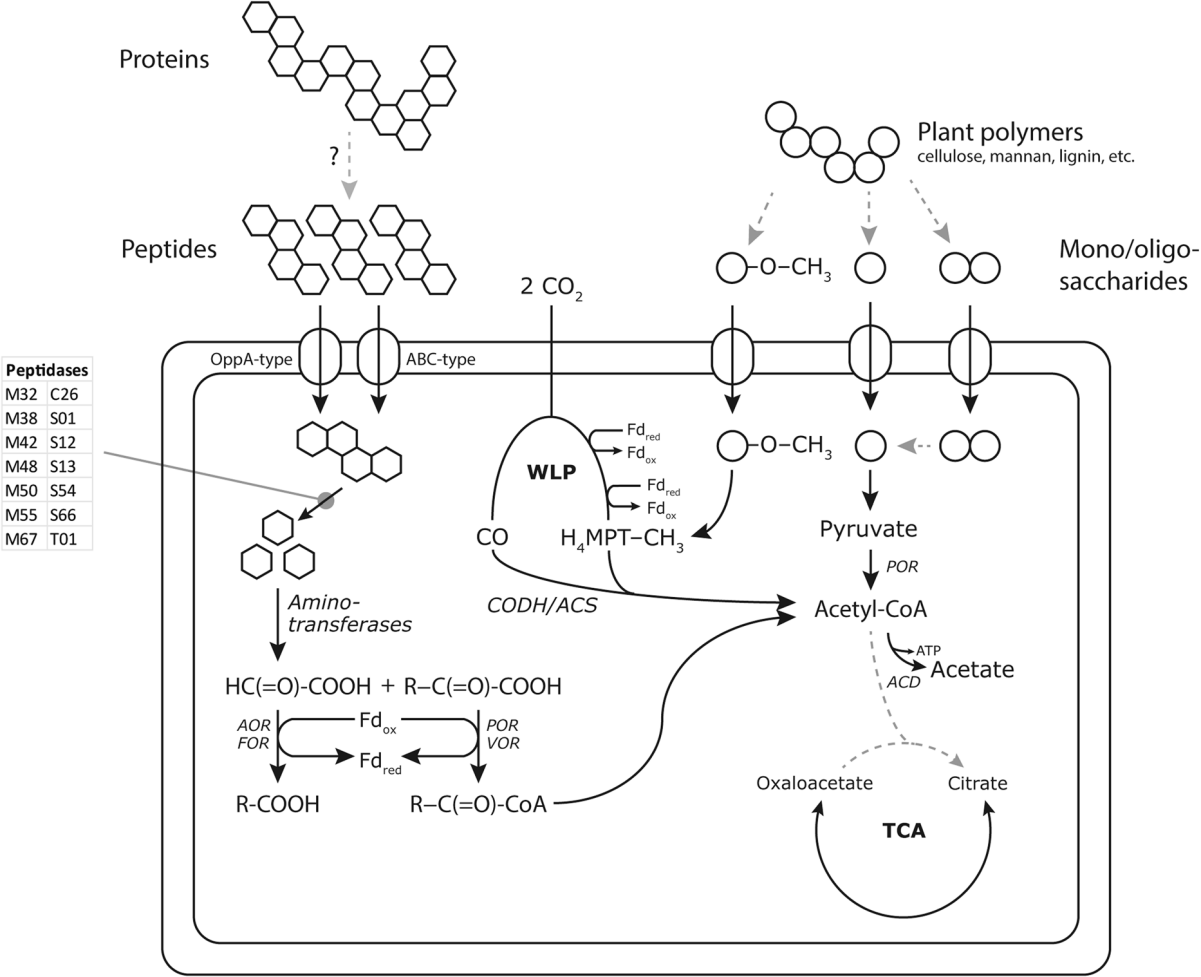
Schematic overview of the central metabolism of the Polar Fox Lagoon Bathyarchaeota. Peptide fermentation was the most complete route for generating reduction equivalents in all MAGs, whereas only partial plant polymer degradation pathways were detected. Potential for acetogenesis was encoded in a minority of MAGs and methanogenesis appeared to be completely absent due to the lack of the methyl-CoM reductase complex. AOR: aldehyde:ferredoxin oxidoreductase; POR: pyruvate:ferredoxin oxidoreductase; VOR: 2-oxoacid:ferredoxin oxidoreductase; WLP: Wood-Ljungdahl Pathway; TCA: tricarboxylic acid cycle; ACD: acetyl-CoA synthase

An early publication on Bathyarchaeota by Lloyd et al. (2013) based on single-cell genomics analyses predicted that these micro-organisms are capable of conserving energy from protein degradation. They showed the presence of genes for both intra- and extracellular peptidases, as well as aminotransferases and various donor:ferredoxin oxidoreductases (Fig. 1). We mined the PFL MAGs for putative peptidase genes by blasting coding sequences against (a subset) of the MEROPS database (Rawlings et al. 2018) and used SignalP 5.0 (Almagro Armenteros et al. 2019) to predict the location of annotated proteins. The results of these analyses are summarised in Supplementary table S2.

The MAGs contained an average of 29 peptidase sequences per genome, with a range of 11 (MAG137) to 57 (MAG019). MAG137 had the lowest completeness of all the PFL MAGs (55%) and this likely led to an underrepresentation of the true number of peptidases it encoded. Lower peptidase counts were not correlated with lower completeness or vice versa. This finding implies that some of the PFL Bathyarchaeota have access to a broader array of peptide-degradation machineries. Peptidases corresponding to all catalytic types were detected, with metallopeptidases (M family) being the most abundant. A number of protease families have highly specialized functions, such as A31, a HypD hydrogenase maturation peptidase that is present in all MAGs except MAG060, MAG117, MAG125 and MAG137. Other (nearly) ubiquitous peptidases include the archaeal proteasome complex (T01), a rhomboid-type peptidase which is predicted to cleave substrates inside the membrane bilayer (S54), chymotrypsin (S01), an isopeptidase that cleaves ubiquitin from peptides (M67), and several endo-, exo- and omega peptidases that have broad specificity (M32, M38, M42, M48, M50 and C26). This combination of peptidases suggests these Bathyarchaeota may degrade a wide range of proteins. Seven of the MAGs encode for peptidases specific to D-Ala, which is found in bacterial cell walls and as an osmolyte in certain invertebrates: M55, S12, S13 and S66. MAG107 appears to be specialized towards this metabolism, encoding all four families and S12 in three copies. Nine MAGs encode a collagenase (U32), which is not typically found in archaea, but rather in pathogenic bacteria such as *Helicobacter pylori* and *Escherichia coli*.

None of these peptidases are classified into groups known to be exported to the extracellular space (Maupin-Furlow 2018) and correspondingly, SignalP 5.0 (Almagro Armenteros et al. 2019) only identified export signal peptides in seven sequences in total. These are the M14 carboxypeptidase of MAG011 (also annotated in MAG060 and MAG107), the S08 endopeptidase of MAG011 (also detected in MAG019 and MAG107), both G01 endopeptidases in MAG033 and MAG097, the S12 D-Ala/D-Ala carboxypeptidase of MAG097 (three copies detected in MAG107), signal peptidase S26 of MAG117 (found in multiple MAGs, see supplementary tables), and the rhomboid peptidase S54 of MAG125 (detected in all MAGs, except MAG095, MAG107 and MAG121).

Many MAGs encoded (several) oligopeptide transporters (Fig. 1). Of these the OppA-type transporter initially described in *Salmonella typhimurum* was the most prevalent, occurring in 12 of the 19 MAGs (not in MAG066, MAG095, MAG097, MAG102, MAG117, MAG121, MAG125 and MAG137). The other transporters are solute-binding protein subunits of ABC-type transporters (Nelson et al. 1999), whose physiological substrates are difficult to predict from sequence information alone. We did not detect specific protein transporters in MAGs with numbers of 66 and up, which may be due to their lower genome completeness (< 85%). All MAGs encoded several genes for aminotransferases. Aside from anabolic aminotransferases involved in the synthesis of UDP-N-acetyl-D-glucosamine, a precursor for peptidoglycan synthesis, the main classes were aromatic amino acid aminotransferases, histidinol-phosphate aminotransferase, and acetylornithine aminotransferase.

Degradation of the amino acid backbone is predicted to proceed through one of several donor:ferredoxin oxidoreductases, yielding reduced ferredoxin as the final product. These include aldehyde:ferredoxin oxidoreductases present in all MAGs (possible substrates include formaldehyde, acetaldehyde, 1-propanal and 1-butanal), with some MAGs containing an additional formaldehyde-specific oxidoreductase (MAGs 004, 011, 014, 019, 033, 048, 060, 098, 107 and 125), 2-oxoacid:ferredoxin oxidoreductase (MAGs 002, 011, 014, 019, 033, 060, 062, 066, 095, 102, 107 and 114) and pyruvate/ketoisovalerate:ferredoxin oxidoreductase (all MAGs except 060 and 137). In the hyperthermophilic archaeon *Pyrococcus furiosus*, aryl pyruvates produced by the transamination of aromatic amino acides can be oxidized by indolepyruvate:ferredoxin oxidoreductase (IOR), yielding additional reduced ferredoxin (Mai and Adams 1994), however, none of the PFL MAGs encoded this gene. Acetyl-CoA formed as a result of amino acid oxidation can be converted to acetate by acetyl-CoA synthase, a reaction which yields one mole of ATP per acetyl-CoA through substrate-level phosphorylation. Archaea have so far not been shown capable of performing the reductive Stickland reactions, instead they rely on the reduction of elemental sulphur to sulphide or the formation of hydrogen to maintain redox balance (Schönheit et al. 2016). Blast searches did not detect sulphur reductases in any of the MAGs. The presence and predicted functions of hydrogenase genes is discussed in more detail below.

### Polar Fox Lagoon Bathyarchaeota show limited potential to degrade carbohydrate polymers and aromatic compounds

Some previously studied Bathyarchaeotal genomes showed a capacity for the degradation of plant-derived polymers, such as (hemi-)cellulose, mannan, starch, pectin and lignin (Fig. 1). Recently, enrichment cultures using these compounds as substrates have supported the genome-based results from metagenomic studies (Hu et al. 2021; Yu et al. 2018). We mined the PFL Bathyarchaeotal MAGs for carbohydrate-active enzymes (CAZymes) using the dbCAN2 server (Yin et al. 2012; Zhang et al. 2018), including annotation of putative CAZyme gene clusters with CGC-finder (CAZyme Gene Cluster). These results are shown in Supplementary table S3. The dbCAN2 server uses three tools to annotate CAZymes (HMMer, DIAMOND and eCAMI) and recommends only considering hits that are recognized by at least two of these. However, because the Bathyarchaeota are a deeply branching lineage, which may provide an obstacle to correct annotation, below we also discuss results based on annotation by HMMer alone.

The dbCAN2 annotation resulted in the annotation of alpha-amylase (GH57) in 11 out of 19 MAGs, indicating the ability to degrade starch in these organisms, yielding a mixture of maltose and dextrins. However, only 7 MAGs encoded the glucosidase enzyme (GH133) required to further break down these di- or oligosaccharides to monosaccharides which can be fed into central carbon metabolism. Both categories are strongly supported by annotation by all three of the tools used.

Other polymer degradation CAZyme categories that were annotated in a majority of the MAGs included AA6, 1,4-benzoquinone reductases, involved in aromatic compound degradation; GH1, a family of β-sugar hydrolases; GH109, α-N-acetylgalactosaminidase, involved in glycoprotein deglycosylation; GH5 and GH130, a β-mannanase and β-1,4-mannosylglucose phosphorylase, involved in β-mannan breakdown; and CBM50, representing a family of LysM-domain peptidoglycan-binding proteins. The annotation for these families was weakly supported, in many cases only by HMMer results. Recently, the enzymes involved in the metabolism of methoxylated aromatic compounds in the methanogenic archaeon *Methermicoccus shengliensis* were identified (Kurth et al. 2021), enabling the convenient screening of (meta)genomic sequences for their presence. We conducted a manual BLASTP search for these genes involved, MtoABCD. Of these four, MtoB (the *O*-demethylase that releases a methyl group from lignin) and MtoC (the corrinoid protein that accepts the methyl group from MtoB) are crucial (Kurth et al. 2021). The results are summarized in Table 3, which shows that only four MAGs encode both MtoB and MtoC. Of these, MAGs 048, 060 and 107 also encode MtoA and MtoD. MAGs 014 and 062 lack only a gene for MtoB, which may be a result of the incompleteness of the metagenomic bins. These results show that the degradation of methoxylated aromatic compounds by Bathyarchaeota is possible in the Polar Fox Lagoon sediment (Fig. 1). However, the fate of the methyl group transferred from the methoxylated substrate is unknown, as a methanogenesis pathway was found to be lacking in these genomes (see below).

**Table 3:** BLASTP hits for enzymes involved in the liberation of methyl groups from methoxylated aromatic compounds (such as lignin). Empty cells represent sequences for which no significant hits were found

MAG	MtoA (%)	MtoB (%)	MtoC (%)	MtoD (%)
MAG002			28	38
MAG004			40	43
MAG011			31	
MAG014	49		36	46
MAG033			34	
MAG048	49	36	37	44
MAG060	34	33	47	42
MAG062	38		37	35
MAG066	39		30	
MAG095			30	
MAG097	38		40	
MAG098				
MAG102	37		32	
MAG107	42	43	53	37
MAG114	38	25	39	
MAG117				
MAG121				
MAG125				
MAG137				

In addition to carbohydrate degradation pathways, several carbohydrate synthesis enzymes were annotated by dbCAN2. These included: GT2, which contains enzymes involved in, for example, cellulose and chitin synthesis; GT4, whose only archaeal member was shown to synthesize trehalose from nucleoside sugars in *Pyrococcus horikoshii* (Ryu et al. 2005); and GT5, which contains glycogen synthases. It is unclear what the function of the GT2 family proteins could be, especially given that the reactions catalysed are favoured in the synthesis direction according to Biocyc (Karp et al. 2019). Trehalose potentially has a role in osmoadaptation and several archaea are known to use trehalose itself and derivatives such as 2-sulfotrehalose as a compatible solute (Roberts 2004). Sucrose can also act as a compatible solute, but in archaea it is typically imported rather than synthesized (Roberts 2004). In previous work, it was shown that the addition of small amounts of sucrose can completely inhibit trehalose synthesis in *Natronococcus* sp. (Desmarais et al. 1997). With the inflow of salt water from Tiksi Bay expected to increase in the future as the Polar Fox Lagoon becomes fully exposed, organisms which are capable of mitigating osmotic stress may have a distinct advantage over those that do not.

Annotation with CGC-finder did not reveal CAZyme gene clusters in any of the MAGs, although a false negative result here cannot be ruled out due to the fragmented nature of the genomes (the lowest number of contigs is 117 for MAG019). Strongly linked gene clusters, complete with transcription factors and transporters, can be detected in highly specialized carbohydrate-degrading organisms, such as gut Bacteroides (Huang et al. 2018). While the absence of these clusters in the Bathyarchaeotal MAGs does not rule out carbohydrate utilization by these organisms, it means that it is less likely that these pathways contribute to energy conservation in the PFL Bathyarchaeota.

### PFL Bathyarchaeota do not have methane metabolism, and only a few are predicted to be able to produce acetate

Recent research has shown that some Bathyarchaeotal genomes contain genes encoding a complete pathway for methane production (Evans et al. 2015). McrA is the alpha subunit of the methyl-coenzyme M reductase complex, which catalyzes the final step of methanogenesis (or, conversely, the first step of anaerobic methane oxidation). Divergent Mcr enzymes were also found to perform the oxidation of higher chain alkanes (Borrel et al. 2019; Laso-Pérez et al. 2019, 2016; Wang et al. 2021, 2019). Neither BLASTP nor HMMER (using the sequences provided by Evans et al. as query) were able to detect the presence of McrA in any of the PFL MAGs, showing that this is likely not a metabolism used by these organisms.

(Homo)acetogenesis was suggested as another potential lifestyle based on genomic data (He et al. 2016). Production of acetate from two molecules of CO_2_ proceeds through the Wood-Ljungdahl pathway (WLP), using either tetrahydrofolate (in bacteria) or tetrahydromethanopterin (in archaea) as the C1 methyl group carrier (Ragsdale 2008). In archaea, the requisite electrons are provided by reduced ferredoxin (for the formation of formylmethanofuran and carbon monoxide from CO_2_) and F_420_H_2_ (twice, for the reduction of CH = H_4_MPT to CH_2_ = H_4_MPT and finally to CH_3_-H_4_MPT), see Fig. 1. The key enzyme in this pathway is the carbon monoxide dehydrogenase/acetyl-CoA synthase (CODH/ACS) complex. Of the nineteen MAGs only six (033, 048, 060, 097, 107 and 114) contained genes for a full WLP. Acetogenesis may function as a redox sink, recycling reduced ferredoxin produced by peptide/amino acid degradation. However, it is unknown how the F_420_ required for these reactions is synthesized, as we did not detect the genes required for the known F_420_ biosynthesis pathway. The potential to form acetate is not linked to a specific Bathyarchaeotal group, as the WLP-positive MAGs are distributed across all orders classified in this population by GTDB.

### Hydrogenases are unlikely to be used as redox sinks by Polar Fox Lagoon Bathyarchaeota

**Fig. 2:**
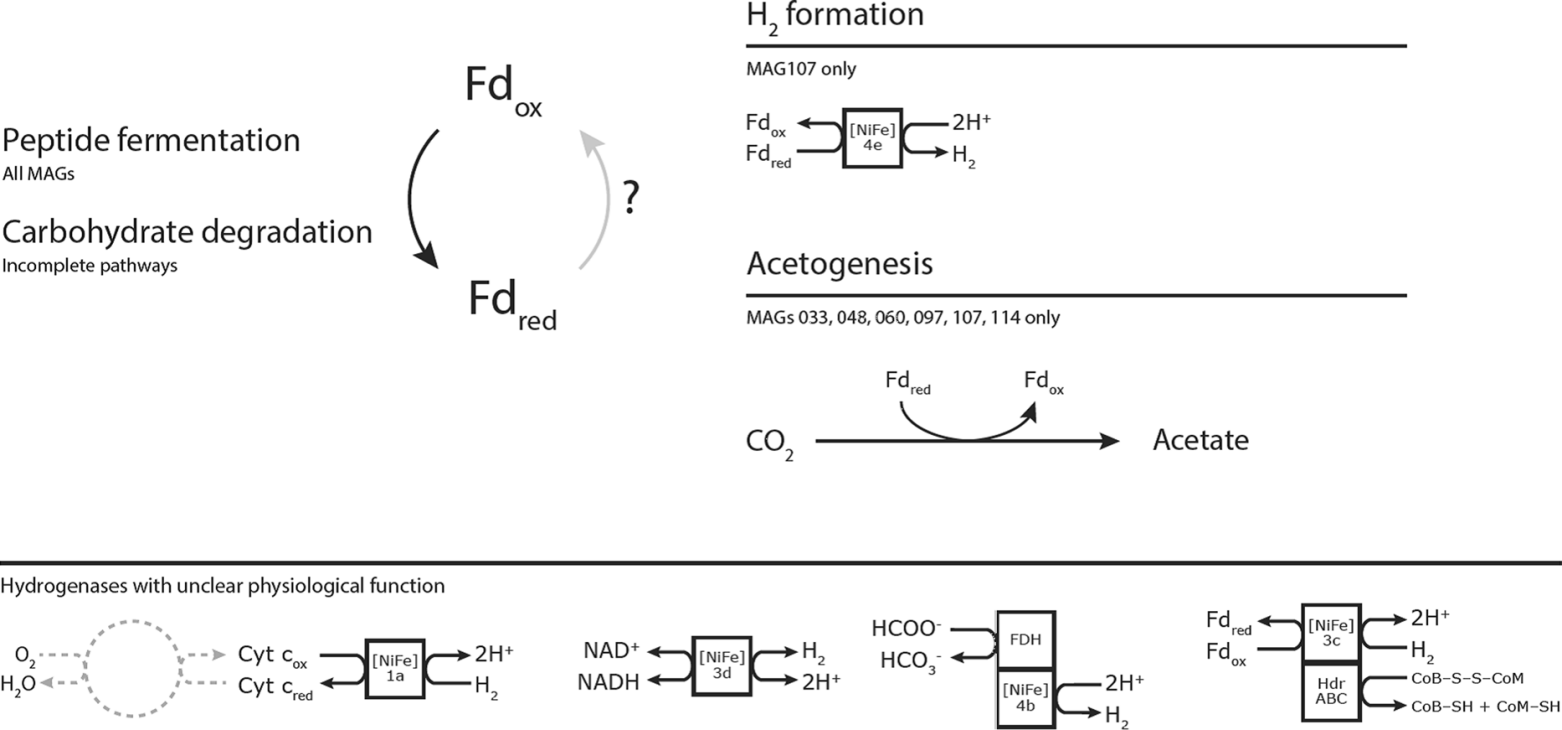
Illustration of the lack of information regarding redox balance in the Polar Fox Lagoon Bathyarchaeota. Hydrogen formation from ferredoxin is limited to a single genome; acetogenesis is restricted to six. MAG: metagenome-assembled genome; Fd_ox/red_: oxidized/reduced ferredoxin; Cyc *c*_ox/red_: oxidized/reduced cytochrome *c*; FDH: formate dehydrogenase; Hdr: heterodisulfide reductase

Fermentative organisms require a way to recycle reduced electron carriers, such as NADH and ferredoxin. Bathyarchaeota are predicted to generate ferredoxin from the degradation of, for example, peptides through (form)aldehyde:ferredoxin oxidoreductases and pyruvate:ferredoxin oxidoreductases. Given that these genomes do not suggest the capacity to generate methane, and that acetogenesis appears to be limited to only a few of the PFL Bathyarchaea, a reasonable hypothesis is that they recycle reduced ferredoxin through hydrogen (H_2_) production. There are several classes of hydrogenases that are capable of evolving H_2_ using Fd_red_ as the electron donor, namely [NiFe]-type energy-conserving hydrogenases (Ech) (Schoelmerich and Müller 2020) and [FeFe]-type hydrogenases. The latter group was not detected in any of the PFL genomes by either DRAM or manual BLASTP searches. The [NiFe]-type hydrogenases are a diverse family of enzyme complexes that have various physiological roles and have been classified into groups accordingly (Søndergaard et al. 2016). DRAM annotated only 14 catalytic hydrogenase subunits across all MAGs (Table 4, Fig. 2), ten of which are classified as 3c. This group contains Mvh-type hydrogenases, which form a complex with and provide reducing equivalents to heterodisulfide reductase, which recycles CoB and CoM in methanogenesis as well as reduces ferredoxin (Wagner et al. 2017). The remaining hydrogenases belong to group 1a, a group of unidirectional H_2_-uptake hydrogenases; 3d, which contains NAD-dependent bidirectional hydrogenases; 4b, which represents formate-respiring hydrogenase complexes; and finally, 4e, the only type of ferredoxin-utilizing hydrogenase annotated in the PFL MAGs (MAG107).

**Table 4:** HydDB classification of catalytic hydrogenase subunits detected in the Bathyarchaeotal MAGs

MAG004	[NiFe] Group 3c	Heterodisulfide reductase-linked
MAG033 (2x)	[NiFe] Group 3c	Heterodisulfide reductase-linked
MAG062	[NiFe] Group 3c	Heterodisulfide reductase-linked
MAG066	[NiFe] Group 4b	Formate-respiring
MAG095 (3x)	[NiFe] Group 3c	Heterodisulfide reductase-linked
MAG098	[NiFe] Group 3c	Heterodisulfide reductase-linked
MAG107	[NiFe] Group 4e	Ferredoxin-coupled, Ech-type
MAG114	[NiFe] Group 1a	Respiratory H_2_-uptake
MAG117	[NiFe] Group 3d	NAD-coupled
MAG125	[NiFe] Group 3c	Heterodisulfide reductase-linked

The physiological role of most of the annotated hydrogenases in these Bathyarchaeota is unclear. The Mvh-type group 3c hydrogenases are not necessary for either CoB/CoM recycling (due to the lack of a complete methanogenesis pathway) or reduced ferredoxin production through flavin-based electron bifurcation (reduced ferredoxin is produced through peptide fermentation). The group 1a uptake hydrogenases require a cytochrome subunit that functions as an electron acceptor (Søndergaard et al. 2016), which was not detected in MAG114. Previous research established that cytochrome *c* and its corresponding O_2_-consuming oxidase complex can have a role in oxygen detoxification (Ramel et al. 2013). It is possible that the cytochrome *c* subunit was not detected due to the incompleteness of the genome but given that MAG114 does not encode a cytochrome oxidase complex either (Fig. 2), the terminal electron acceptor of this complex and its function remain unknown.

### Redox balancing through syntrophy is not possible for a majority of PFL Bathyarchaeota

Syntrophy requires the exchange of electrons between two or more microbial species, for which several mechanisms are known, such as hydrogen or formate formation (Sieber et al. 2012), through soluble quinone exchange (Smith et al. 2015) and direct interspecies electron transfer through conductive pili (Lovley 2017).

We have established in the previous section that the formation of hydrogen is an unlikely route for redox balancing in the PFL Bathyarchaeota. The generation of formate (from CO_2_) proceeds through formate dehydrogenase (FdhAB), a two-subunit enzyme that uses NADH as electron donor. We detected genes encoding both subunits in MAGs 014, 033, 060, 066, 107 and 114; and genes for only one subunit in MAGs 002, 011, 019, 048, 097, 102 and 137. Additionally, because NADH is the electron donor for this reaction, either a bifurcating [FeFe]-type hydrogenase, or the Rnf membrane complex is required to perform the reduction of NAD^+^ using reduced ferredoxin. No [FeFe]-type hydrogenases were detected in our metagenome, making this route unlikely. Of the six subunits of RnfABCDEG, blast searches only found RnfB (18 out of 19 MAGs, not in MAG137), RnfC (4 out of 19 MAGs: 097, 107, 114 and 121) and RnfD (3 out of 19 MAGs: 033, 097 and 137). Recent experimental work has demonstrated that RnfB is the site of ferredoxin oxidation and RnfC the site of NAD^+^ reduction, but it is unclear whether the RnfBC complex could function without the other subunits (Kuhns et al. 2020).

We detected no genes for pilin formation in any of the Bathyarchaeotal MAGs and no multi-heme cytochrome complexes that could participate in extracellular electron transfer. Ubiquinone, which is water-insoluble, was the only quinone for which a partial synthesis pathway was detected. It is therefore unlikely that these organisms are capable of any sort of syntrophic metabolism through currently known mechanisms.

## Conclusions

We have presented an analysis of the metabolic potential encoded by 19 MAGs classified as Bathyarchaeota recovered from the Polar Fox Lagoon, a Siberian thermokarst lake with a seasonal connection to the brackish Tiksi Bay. In the past, metagenomic and single-cell sequencing data on Bathyarchaeota showed the metabolic potential to participate in the global carbon cycle through, for example, heterotrophic degradation of complex polymers, methanogenesis and acetogenesis. The MAGs recovered from the Polar Fox Lagoon samples revealed the potential for peptide/protein degradation, but limited capacity for (plant-derived) carbohydrate consumption. Methanogenesis (or, conversely, methane/alkane oxidation) is not a likely lifestyle for these organisms, as the key enzyme Mcr/Acr is missing in all 19 MAGs. Only a few MAGs encoded the CODH/ACS complex that is essential for acetogenesis through the Wood-Ljungdahl pathway. Several questions remain unanswered however, for example: (1) How is protein degradation initiated with the apparent lack of extracellular peptidases? (2) What is the role of the limited number of CAZymes in these organisms, given that the breakdown of complex carbohydrates is a stepwise process and many downstream enzymes were not detected? (3) How is redox balance achieved in the organisms that lack both ferredoxin-coupled hydrogenases and the potential to perform either acetogenesis or syntrophy?

Definitive answers to these questions can only be obtained through physiological experiments and/or integrated multi-(meta)omics approaches, both of which require additional sampling campaigns. Future experiments can take advantage of recent progress in the enrichment of Bathyarchaeota reported by Hu et al (2021). Additionally, it will be of great interest to follow the community composition in the Polar Fox Lagoon sediments as it transitions into being fully connected to Tiksi bay, with the accompanying changes in salinity and water chemistry, and to unravel the influence of changes in environmental conditions on the different Bathyarchaeotal subgroups.

## Supplementary Information

Below is the link to the electronic supplementary material.

Supplementary Table S1: Non-Bathyarchaeotal MAGs recovered from Polar Fox Lagoon metagenome (XLSX 29 KB)

Supplementary Table S2: MEROPS classification of putative peptidases (XLSX 14 KB)

Supplementary Table S3: dbCAN2 annotation results (XLSX 15 KB)

## Data Availability

All data related to this manuscript is publicly available through the NCBI BioProject database using accession number PRJNA821074.
